# Effect of Different Kefir Source on Fermentation, Aerobic Stability, and Microbial Community of Alfalfa Silage

**DOI:** 10.3390/ani11072096

**Published:** 2021-07-14

**Authors:** Fisun Koç, Emel Özkan Ünal, Berrin Okuyucu, Selim Esen, Raziye Işık

**Affiliations:** 1 Department of Animal Science, Tekirdag Namık Kemal University, Tekirdag 59030, Turkey; fkoc@nku.edu.tr (F.K.); ozemel@nku.edu.tr (E.Ö.Ü.); berrinokuyucu25@hotmail.com (B.O.); 2 Balikesir Directorate of Provincial Agriculture and Forestry, Republic of Turkey Ministry of Agriculture and Forestry, Balikesir 10470, Turkey; 3 Department of Agricultural Biotechnology, Tekirdag Namık Kemal University, Tekirdag 59030, Turkey; risik@nku.edu.tr

**Keywords:** kefir, alfalfa, silage, fermentation quality, microbial communities

## Abstract

**Simple Summary:**

Minimizing silage additives cost while increasing silage quality is important for a sustainable livestock enterprise, especially in undeveloped and developing countries. In this study, therefore, commercially available kefir yeast (CK) and homemade kefir culture (HK), as a low-cost additive, was applied at untreated a common control (CON) and three different application doses (5.0, 5.7, and 6.0 log cfu g^−1^) on wilted alfalfa and evaluated with the fermentation characteristics and aerobic stability. The addition of HK with an application dose greater than 5.0 log cfu g^−1^ prevents mold formation and inhibits yeast counts in silages. Indeed, both CK and HK improve the silage quality and aerobic stability of alfalfa even with low water-soluble carbohydrate content.

**Abstract:**

The present study has been one of the first attempts to thoroughly examine the effects of different kefir sources on fermentation characteristics, aerobic stability, and microbial communities of alfalfa silages. The effects of commercial kefir (CK) and homemade kefir culture (HK) applied with untreated a common control (CON) and three different application doses (5.0, 5.7, and 6.0 log cfu g^−1^) on wilted alfalfa and stored at an ambient temperature of 25–30 °C are studied. After 45 days of ensiling, fermentation characteristics and aerobic stability of silages were measured, and bacterial diversity was investigated by 16S ribosomal RNA gene sequencing using the GenomeLab™ GeXP platform. Both CK and HK accelerate more lactic acid production and reduced ammonia nitrogen concentration. Factor analysis of kefir sources suggests that the addition of kefir improves the aerobic stability of silages, even the initial water-soluble carbohydrate (WSC) content is inadequate via its antimicrobial effect on yeast and mold formation. *Enterococcus faecium*, *Pediococcus pentosaceous* and *Lactobacillus brevis* were dominant bacterial species among the treated groups at silo opening, while *Lactobacillus plantarum* and *Lactobacillus brevis* became dominant bacterial species after 7 days of aerobic exposure. In conclusion, the application of kefir on alfalfa silages improves fermentation quality and aerobic stability even with low WSC content.

## 1. Introduction

Alfalfa (*Medicago sativa* L.), one of the most common perennial forage legumes, plays a pivotal role in meeting the nutritional requirements of ruminants worldwide. It is mainly associated with high protein content and high nutritional quality, yielding high dry matter per acre and broader adaptation capability [1]. The most well-known application to minimize nutrient losses for future use of fresh forage crops is ensiling. Nevertheless, the main challenge faced by many researchers at ensiling process is the high buffering capacity (Bc) and low concentration of water-soluble carbohydrates (WSC), and high moisture content of alfalfa which results in undesirable secondary clostridial fermentation [2]. In such cases, the use of inhibitors or inoculants becomes mandatory to dominate microbial biota by lactic acid bacteria (LAB), which fermented the lysed plant membranes to lactic acid resulting in a lower pH of the ensiled material [3].

Commercial LAB has been used for many years as microbial additives owing to their inhibitory effect on undesirable microorganisms, e.g., *Clostridia*, *Enterobacter* and several other bacteria [4]. It is also well known from previous studies that LAB has a favorable contribution to the quality of the silage in terms of flavor and sensory profile, and preservation time of the final products [5,6]. However, as previously stated by Tao et al. [7], adding commercial LAB is one of the most frequently stated problems due to its high cost, especially in undeveloped and developing countries. Schnürer and Jonsson [8] were obviously right to draw our attention to the ingredients of an excellent starter culture for well-preserved silage, recommending a combination form of yeast and LAB; hence, kefir might be used as an alternative silage additive due to its complex symbiotic diversity of microorganisms, which has shown heterofermentative properties including LAB, yeast, and acetic acid bacteria [9]. Furthermore, kefir grains are recognized as a unique natural dairy starter culture that can be used several times, and kefir-derived products are also known as easily prepared, low-cost, and fascinating functional food with health benefits [10,11]. Kefir has also received considerable scholarly attention in recent years due to its antioxidant, anti-inflammatory, antifungal, antibacterial specialties, and various pharmaceutical attributes [12,13,14]. Previous research evaluating the effect of kefir microorganisms on aerobic spoilage of sorghum and wheat-straw silages also strengthens these assumptions [15,16]. However, there is a notable lack of high-quality research focusing specifically on fermentation characteristics, aerobic stability, and the microbial community of alfalfa silages inoculated with different kefir sources.

The aim of this paper was to evaluate the effectiveness of different kefir sources, which are produced commercially or homemade, and their various application doses onto first-cut alfalfa for improving fermentation characteristics and aerobic stability. The specific objective of this study was to determine the microbial community of silages by high-throughput sequencing methods after ensiling and aerobic exposure (AE) to explain the impact of isolated bacteria from alfalfa silages.

## 2. Materials and Methods

### 2.1. Forage and Silage Preparation

The plant material of the current study, alfalfa, was grown in an experimental plot of the Field Crops Department of Namık Kemal University (40.59° N and 27.34° S, Tekirdag, Turkey). The total precipitation, long-term mean precipitation, mean average temperature, and long-term mean average temperature of the experimental year was 299.0 mm and 581.5 mm, 15.7 °C and 14.0 °C, respectively. Since the total precipitation was not sufficient, forage water demands were supplied by irrigation fortnightly. First-cut alfalfa was harvested at the early blooming stage (10–20%) on 18 May 2019, by a forage harvester, wilted for 24 h, and manually chopped to approximately 1.5–2.0 cm in length. Wilted alfalfa had 304.6 g kg^−1^ dry matter (DM), 202.1 g kg^−1^ DM of crude protein (CP), 15.45 g kg^−1^ DM of WSC, 445 meq NAOH/kg DM of Bc, 7.50 of pH, 5.30 log cfu g^−1^ of LAB, and 8.08 log cfu g^−1^ of yeast and no mold before ensiling.

The silage was made in a laboratory-scale fermentation system: approximately 500 g wilted alfalfa was weighed and packed into polythene bags and then sealed by a vacuum sealer (CAS CVP−260PD). A commercial kefir yeast (MYStarter KF, contains *Lactococcus lactis* subsp. *lactis* biovar diacetylactis, *Lactobacillus brevis*, *Leuconostoc mesenteroides* subsp. *mesenteroides* ve *Saccharomyces cerevisiae* strains) and homemade kefir culture (contains *Enterococcus faecalis*, *Lactobacillus brevis* and *Micrococcus luteus* according to the 16S rRNA gene sequencing) was used for comparison. The silage treatments (each, 10 replicates) were designed as commercial kefir yeast (CF), and homemade kefir culture (HK) with an untreated common control (CON) and three different application doses (5.0, 5.7, and 6.0 log cfu g^−1^ of fresh matter) for 45 d of ensiling at an ambient temperature of 25–30 °C.

### 2.2. Chemical Analysis

After 45 d of ensiling, opened silos subsampled for microbial enumeration and aerobic stability determination. A representative 20 g wet silage or pre-ensiled material was taken and gently mixed in 180 mL of distilled water at room temperature for 1 h and then filtered through 4 layers of cheesecloths to determine ammonia nitrogen (NH_3_-N) and organic acid content. The pH of each silo was measured using silage extract with a pH meter (WTW-inoLab ph 730). The DM of samples was determined by drying at 60 ± 2 °C in an air-forced oven for 48 h, and DM loss was calculated via the weight differences between wilted alfalfa and opened silage samples. The nitrogen (N) content of wilted alfalfa was measured by the Kjeldahl method and multiplied by 6.25 to get the crude protein (CP) ratio by using AOAC methods. Samples were analyzed for NH_3_-N and WSC as previously reported by Anonymous [17] by using micro distillation and 0.2% anthrone reagent, respectively. The Bc of pre-ensiled alfalfa was determined by Playne and Mc Donald [18].

The organic acid content of silages (Acetic acid, AA; propionic acid, PA; Butyric acid, BA) was evaluated after deproteinization of silage extract with the metaphosphoric acid-formic acid mixture (3:1, v:v) according to the procedure described by Ulger et al. [19] by using a gas chromatograph (Shimadzu GC−2010+, Kyoto, Japan) with a capillary column (Restek, Bellefonte, PA, USA; 30 m, i. d.: 0.25 mm, f.t.: 0.25 μm), and with flame ionization detector (FID) over a temperature range of 45–230 °C. The lactic acid (LA) content of silages was determined using a spectrophotometric method previously described by Koc and Coskuntuna [20].

### 2.3. Microbial Populations

The LAB, yeast, and mold count was performed by using subsamples immediately after opening the silos and after the 7 d AE according to method described previously by Seale et al. [21]. While the pour plate method and MRS Agar (Merck, Darmstadt, Germany) were used to determine LAB for incubating anaerobically at 30 °C for 3 d, the spread-plate method and potato dextrose agar (Merck, Darmstadt, Germany) were used for yeast and mold enumeration after incubating aerobically at 28 °C for 5 d.

### 2.4. Aerobic Stability Analysis

Based on trapping CO_2_ gases into the KOH solution, the bottle system is one of the most common procedures for determining aerobic stability when reaching thermocouples is difficult. Generally, after 5 to 7 days of AE, pH, CO_2_, LAB, yeast, and mold content of silage are used to assess aerobic stability. In the current study, both the bottle system, previously described by Asbell et al. [22] and thermocouples (HOBO Pendant Temperature/Light 64K Data Logger, Onset Computer Corporation, Bourne, MA, USA) was used to record temperature with a 2 h interval during the 7 d of AE. Aerobic deterioration was considered when the temperature of the silage samples was 2 °C higher than the ambient temperature.

### 2.5. Microbial Diversity Analysis

After the proliferation of LAB in nutrient broth for 16 h, 1000 μL of aliquot was taken and centrifuged at 1000 g for 10 min. Microbial DNA from each silage sample was extracted after opening the silos and after the 7 d AE according to Liu et al. [23]. The 16S rDNA regions were amplified using primers F: 5′-AGAGTTTGATCCCTGGCTCAG−3′ and R: 5′-CCGTCAATTCCTTTGAGTTT−3′ [24]. PCR amplification reactions; 50 µL PCR volumes included: 10 ng rDNA, 1 µM of each primer, 1× PCR Buffer ((NH_4_)_2_SO_4_), 200 µM dNTP, 2.0 mM MgCl_2_ and 0.1U i-Taq™ DNA polymerase (5 U/mL) (iNtRON Biotechnology Inc., Burlington, MA, USA). The cycling protocol was 5 min at 95 °C for initial denaturation, 37 cycles of amplification; 95 °C for 45 s, 60 °C annealings for 60 s, 72 °C for the 40 s, and 10 min at 72 °C for final extension (Applied Biosystems ProFlex PCR System (Applied Biosystems, Foster City, CA, USA)). Afterward, the PCR products were run on 1.0% agarose gel using horizontal electrophoresis, and the gels were stained with SafeView™ Classic (Applied Biological Material Inc., Richmond, BC, Canada). PCR products were visualized under UV light in the Gel Documentation System.

The PCR products were sequenced using GenomeLab™ GeXp Genetic Analysis System (Beckman Coulter, Inc., Fullerton, CA, USA) after the precipitation with 3M NaAc. The chromatogram carefully checked the sequencing of the 16S rDNA region for overlapping nucleotide peaks by using ChromasPro Version 2.1.8 (Technelysium Pty. Ltd., South Brisbane, QLD, Australia). The checked sequences file consisting of MSTN fragments was controlled by the MEGA7 software (Molecular Evolutionary Genetics Analysis, version 7.0) [25]. The sequence data reported in this study were archived in The National Center for Biotechnology Information (NCBI) with the accession numbers MZ014989–MZ015001. The nucleotide sequences of studied 16S rDNA region in different species were performed from The National Center for Biotechnology Information webpage (https://www.ncbi.nlm.nih.gov/, accessed on 12 April 2021). The retrieved partial sequences were aligned by Clustal X, and the phylogenetic tree was generated by the Neighbor Joining (NJ) method (Kimura 2) in MEGA 7 software [25].

### 2.6. Statistical Analysis

Data were previously adjusted for the fixed effects of additive (CK and HK), dose (a common control, 5.0, 5.7, and 6.0 log cfu^−1^), and the interaction between these effects. The adjustment was made by analysis of variance using procedure PROC MIXED from SAS [26], considering the following statistical model:

Yijk = μ + ai + bj + (ab)ij + eijk(1)

in which, Yijk is the value of measured characteristics; μ is a constant associated with each observation; ai is the effect of additive i; bj is the effect of dose j; (ab)ij is the interaction effect between the additive i and dose j; eijk is the random error of each observation. The differences among treatment means were tested using Tukey’s multiple range test, and significance was established at *p* < 0.05. Principal component analysis (PCA) was carried out according to the PRIN method of SAS by using the 19 variables and 1 supplementary variable (treatment group) and only PCAs with eigenvalues higher than 1 were retained and interpreted [19]. Furthermore, obtained PCAs were rotated in orthogonal Varimax rotation by FACTOR procedure of SAS, and only with an absolute loading value higher than 0.50 were considered to load on specific extracted PCAs (SAS, 2004).

## 3. Results

Kefir sources and their application doses did not change the DM, and WSC content of the silages (Table 1). However, comparing the pH and DM loss data reveals that both kefir source and doses significantly affect pH (*p* < 0.05), and the DM loss values (*p* < 0.001) of silages. In addition, the HK with an application dose of 6.0 log cfu g^−1^ (26.4 g kg^−1^ DM), CON (26.2 g kg^−1^ DM), and CK with an application dose of 5.0 log cfu g^−1^ (26.2 g kg^−1^ DM) presented higher DM loss. On the other hand, no significant results were obtained for WSC content among the silage groups (*p* > 0.05).

**Figure 1:**
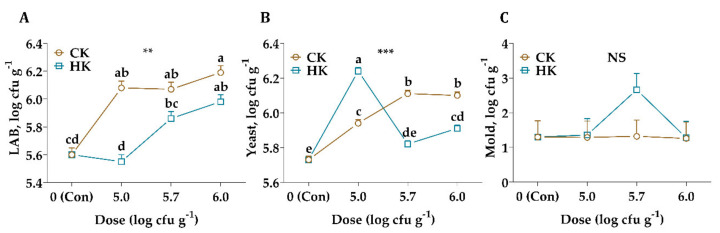
Changes in lactic acid bacteria (LAB, (**A**)), yeast (**B**), and mold (**C**) count of alfalfa silage after 45 d of ensiling. CK: commercial kefir, HK, homemade kefir, cfu: colony-forming units. The values with different letters (a, b, c, d) in each graph are statistically different (*p* < 0.05), NS: not significant, ** : *p* < 0.01, *** : *p* < 0.001.

A comparison of the microbiological composition of alfalfa silages after silo openings reveals that the highest LAB count was observed in the CK group with an application dose of 6.0 log cfu g^−1^ (*p* < 0.01, Figure 1). Besides, from the figure above we can see that yeast count was affected significantly from different kefir sources (*p* < 0.001), while the mold count was not (*p* > 0.05).

**Figure 2:**
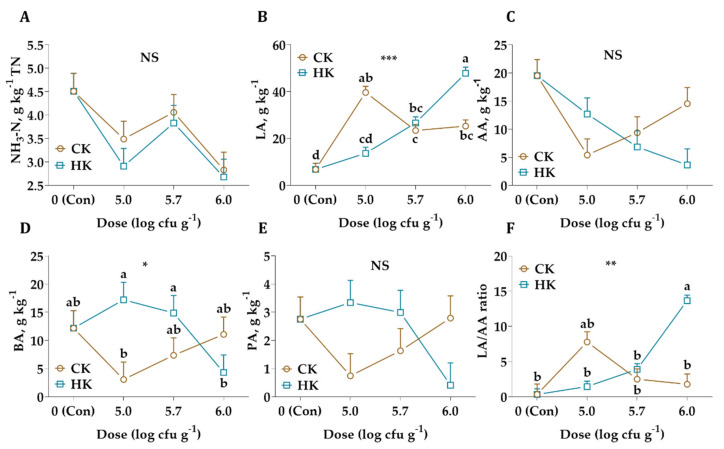
Changes in ammonia nitrogen (NH_3_-N, (**A**)), lactic acid (LA, (**B**)), acetic acid (AA, (**C**)), butyric acid (BA, (**D**)), propionic acid (PA, (**E**)), and LA/AA ratio (**F**) of alfalfa silages after 45 d of ensiling. CK: commercial kefir, HK, homemade kefir, cfu: colony-forming units. The values with different letters (a, b, c, d) in each graph are statistically different (*p* < 0.05), NS: not significant, * : *p* < 0.05, ** : *p* < 0.01, *** : *p* < 0.001.

From the data in Figure 2, it is apparent that the LA and BA concentrations of silages treated with different kefir sources and application doses were significantly affected (*p* < 0.001 and *p* < 0.05, respectively) while the AA and PA were not (*p* > 0.05). What stands out in this figure is the highest LA/AA ratio observed in the HK group with an application dose of 6.0 log cfu g^−1^, the lowest ratio observed in control. As shown in Figure 2, the results also indicate no statistically significant differences between the kefir sources and their application doses in terms of NH_3_-N concentration (*p* > 0.05).

**Figure 3:**
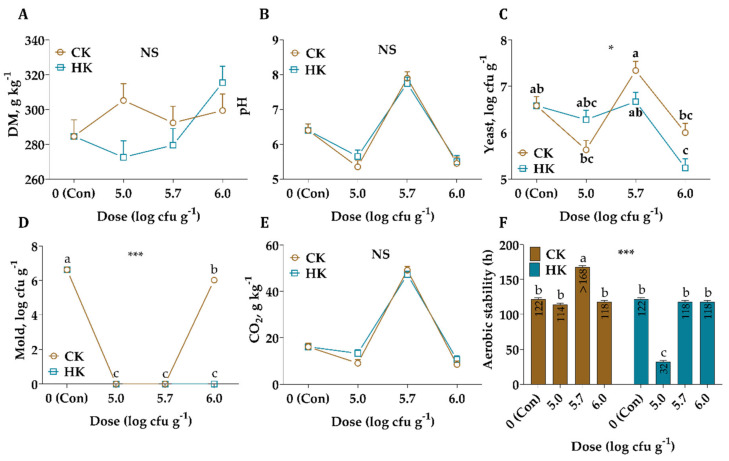
Changes in dry matter (DM, (**A**)), pH (**B**), yeast (**C**), mold (**D**), carbon dioxide (CO_2_, (**E**)), and aerobic stability (**F**) of alfalfa silages after 7 d of aerobic exposure. CK: commercial kefir, HK: homemade kefir, cfu: colony-forming units. The values with different letters (a, b, c) in each graph are statistically different (*p* < 0.05), NS: not significant, * : *p* < 0.05, *** : *p* < 0.001.

The DM, pH, and CO_2_ values were similar among the kefir sources and their application doses, and no significant differences were observed up to 7 d of AE (Figure 3). On the other hand, the differences between the kefir sources and their application doses were significant for yeast (*p* < 0.05) and mold (*p* < 0.001). What is interesting about the data in this figure is that the HK with an application dose of higher than 5.0 log cfu g^−1^ prevents mold formation. Furthermore, a comparison of the data recorded by the data logger reveals that the highest aerobic stability was observed in CK with an application rate of 5.7 log cfu g^−1^ as >168 h while the lowest observed in HK with an application 5.0 log cfu g^−1^ as 32 h (Figure 3).

**Figure 4:**
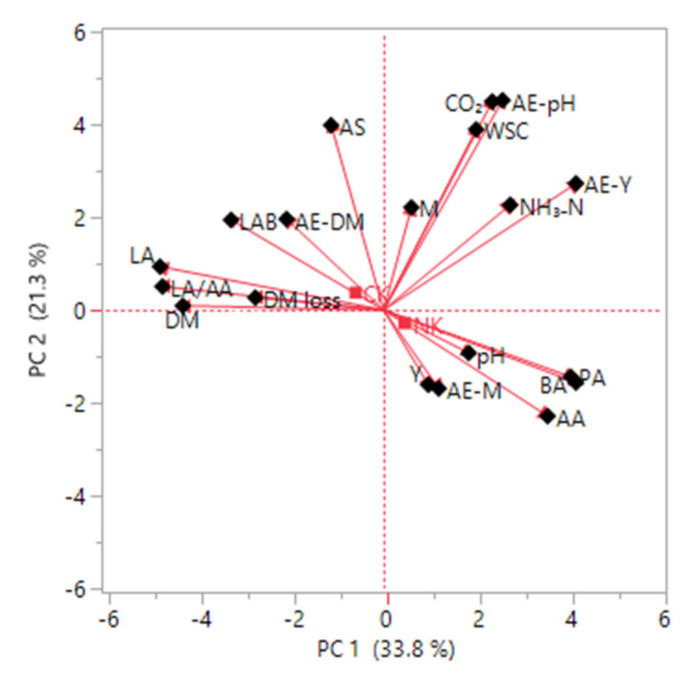
Biplot ordering using principal component analysis of alfalfa silage characteristics inoculated with different kefir sources. CK: commercial kefir, HK, homemade kefir, AA: acetic acid, AE: aerobic exposure, AS: aerobic stability, BA: butyric acid, CO_2_: carbon dioxide, DM: dry matter, DM loss: dry matter loss, LA: lactic acid, LAB: lactic acid bacteria, M: mold, NH_3_-N: ammonia nitrogen, PA: propionic acid, WSC: water-soluble carbohydrate, Y: yeast.

Particularly revealing is how principal factor analysis explains 86.75% of the total variability of original variables (Table 2). Loading vectors associated with the original variables, with eigenvalues higher than 1, are reported and used to interpret the meanings of 5 retained PCs. Biplot ordering using PCA of alfalfa silage characteristics inoculated with different kefir sources is also presented in Figure 4.

As shown in Figure 4, the first 2 components of PCA explain 55.1% of the total variation. Further analysis of the data reveals the variable loadings on PC1 were related to organic acids, such as LA, AA, BA, PA or fermentation characteristics such as NH_3_-N, or a microbiological composition such as LAB and yeast count (after AE), or DM and DM loss. PC1 had an eigenvalue of 6.428 and explained 33.83% of the total variability. While the CO_2_, pH (after AE), WSC, aerobic stability, and yeast count (after AE) loaded on PC2; yeast, DM loss, pH, and mold count (after AE) loaded on PC3 and explained 21.26% and 15.11% of the total variability, respectively. PC4 was characterized by LAB and mold count, and explained 9.92% of the total variability with an eigenvalue of 1.885. The last component, PC5, was characterized by NH_3_-N and pH explaining 6.64% of the total variability with an eigenvalue of 1.261.

**Figure 5:**
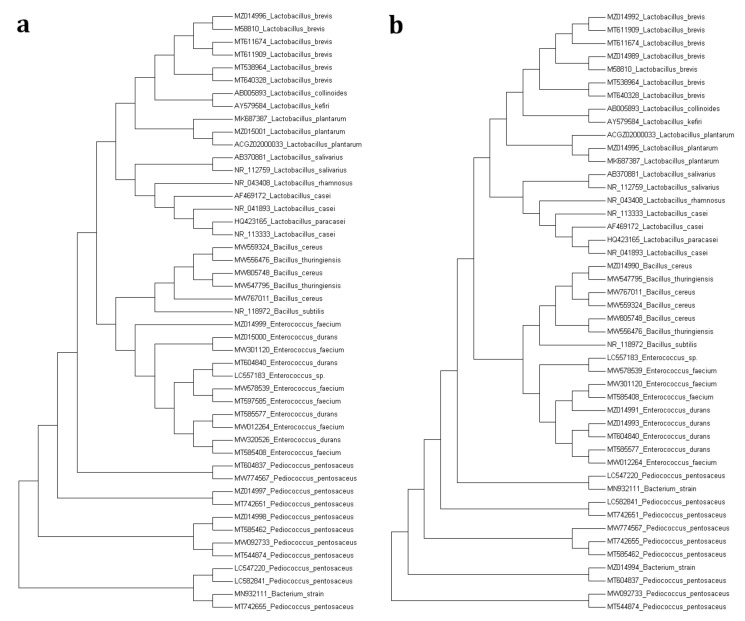
The phylogenic tree of the bacterial community is estimated using the NJ method (Kimura 2) that retrieved sequences provided by the NCBI GenBank database (MZ014989- MZ015001 from this study) (https://www.ncbi.nlm.nih.gov/, accessed on 12 April 2021). (**a**) comparison of sequences that retrieved from NCBI GenBank database and commercial kefir, (**b**) comparison of sequences that retrieved from NCBI GenBank database and homemade kefir.

We performed 16S rRNA sequencing to identify the microbial communities in the alfalfa silages systematically. The predominant bacterial species isolated at silo opening and after aerobic exposure were summarized in Table 3. Besides, the phylogenetic relationship used to estimate the relationship among various species based on genetic distances between the alfalfa silage treated with different doses of kefir sources was shown in Figure 5. As shown in Table 3, most of the bacteria were detected in alfalfa silage treated with different doses of kefir source. Furthermore, 16S rRNA sequence analysis indicates that while *Enterococcus faecium*, *Pediococcus pentosaceous* and *L. brevis* were dominant bacterial species among the treated groups at silo opening; *Lactobacillus plantarum* and *L. brevis* became dominant bacterial species after 7 d of AE.

## 4. Discussion

The present study was designed to determine fermentation quality and microbial community composition of alfalfa silage treated with different kefir sources and to find the most suitable dosage of use. Prior studies have noted the importance of an adequate substrate for LAB, DM, and WSC content to produce stable silages [4,7,27]. The DM content of wilted alfalfa (304.6 g kg^−1^ FW) and LAB concentration (5.30 log cfu g^−1^) before ensiling were consistent with the previous studies [28,29]. However, the WSC of pre-ensiled material (15.45 g kg^−1^ DM) was not adequate, considering the recommendation of 50 g kg^−1^ DM as a minimum required to ensure good fermentation during ensiling [1].

The major limitation of this study is the low WSC content (15.45 g kg^−1^ DM) of wilted alfalfa, which had been not sufficient to initiate lactic acid fermentation (Table 1). One of the most used parameters to determine the level of proteolysis in silages is NH_3_-N. Not significantly, but numerically, the NH_3_-N concentration of alfalfa silages was decreased with the addition of both different kefir sources (Figure 2). Therefore, it seems the Bc capacity of alfalfa plays a more significant role than the proteolysis during fermentation, considering the obtained results of this study. It is a well-known fact that proteolysis could account for more than half of the total N in alfalfa silage during fermentation and may result in efficient N utilization by ruminants [27]. Moreover, it is widely acknowledged that different LAB strains alone or combined with fibrolytic enzymes, sugar source, or organic acid can be used as additives to produce good quality alfalfa silage due to high Bc and low WSC concentration [30,31]. While there is limited information on proteolysis and NH_3_-N formation of kefir treated forage, in our study, the treatment with different kefir source and their various doses conserved more LA (HK produce more LA with an increasing application rate), and reduced NH_3_-N concentration.

Wang et al. [32] stated that one major factor that affects the extent of fermentation is silage pH. The current study found that kefir sources cannot be reduced forages’ pH (all above 5.0) to desired level alone when the initial WSC of forage was inadequate (Table 1). A possible explanation for these results may be the lack of adequate LAB growth in alfalfa silage by accelerating LA production to decrease pH during the fermentation. Prior studies have noted the importance of ensiling of legume forages with high WSC sources to produce more organic acids and obtain better fermentation quality, which are confirmed in our results [33,34].

It is expected that a rapid decrease in pH value with an increased dose of CK and HK inoculated silages. The LAB count was higher in treated silages than in the control silage except for HK with an application rate of 5.0 log cfu g^−1^ (*p* < 0.01, Figure 1). On the other hand, these results were not confirmed by silage pH. This may be explained by the fact that the conversion of LA to BA by undesirable microorganisms in silage, and the growth and metabolism of yeast which is found naturally in kefir fauna, utilized soluble carbohydrates at the initial fermentation stage and resulted in DM losses [35,36].

As previously stated by Kleinschmit and Kung [37], LA/AA ratio could be used as an indicator of an effective homolactic acid fermentation. In general, the study found a tendency for an increasing LA/AA ratio when the application rate was greater than 5 log cfu g^−1^ which means HK serves mainly homolactic characteristics whereas CK serves mainly heterolactic (Figure 2).

Previous research has shown that DM loss reflects the nutritive value and fermentation quality of silage [38,39]. There were no differences in DM contents between alfalfa silages treated with different kefir sources (*p* > 0.05). However, the lower DM loss was observed in silages treated with 5.7 log cfu g^−1^ of CK and 5.0 log cfu g^−1^ of HK. Furthermore, the silages treated with log 5.7 cfu g^−1^ of CK had the highest WSC content compared to other silages. As mentioned in the literature review, the amount of DM loss related to fermentation can vary depending on the dominant microbial species and fermented substrate types [38]. The homolactic LAB found in kefir’s microbial flora played an important role at these application rates to reduce DM loss by limiting plant respiration and undesirable microbial growth. A similar result was also found when chemical additives or homolactic LAB was used as silage additives [40]. Moreover, a tendency to increase DM loss with an increased application rate of HK was detected in this study. This may be related to the increased number of lactate assimilating yeasts in silage, resulting in higher DM losses and aerobic spoilage. A broadly similar point has also recently been made by Bai et al. [36].

Studies to improve feed-out stages of silages show the importance of aerobic stability, due to its potential cause of nutrient and DM loss, leads to health risks in animals and humans in terms of mycotoxins produced from undesirable microorganisms [7,41,42]. Several factors may influence the aerobic stability of silages, such as the size of the LAB population, the composition of inoculants, organic acid concentration, and the WSC content of ensiling material [7]. In this study, the highest aerobic stability was observed in CK with an application rate of 5.7 log cfu g^−1^ according to the indirect measurement of aerobic deterioration (Figure 3). Inconsistent with the expectation, the highest yeast count was observed in CK with an application rate of 5.7 log cfu g^−1^, possibly related to uncontrolled environmental conditions at the initial fermentation stage. On the other hand, the addition of HK with an application dose greater than 5.0 log cfu g^−1^ prevents mold formation and inhibits yeast counts in silages, suggesting that it improve aerobic stability. A possible explanation for this might be that metabolites produced from LAB, such as acetic acid, 1, 2-propanediol, and ethanol, improve aerobic stability [2,42]. Another possible explanation for this is that antimicrobial substances originated from alfalfa silages, e.g., saponin, during the fermentation process possess antimicrobial activity against bacteria and yeast, such as *Bacillus subtilis*, *Candida albicans* and *S. cerevisiae* [43].

The five silage quality components were related to organic acids, and thus, PC1 can be considered an organic acid-type factor. Surprisingly, a positive relationship was found between aerobic stability, pH (after AE), WSC, and yeast count (after AE). This finding was unexpected and suggested that the addition of kefir improves the aerobic stability of silages—even the initial WSC content was inadequate via its antimicrobial effect on yeast and mold formation (PC2, Table 2). The PC3 confirms that DM loss during ensiling is possibly related to the metabolism of yeasts, which utilizes WSC and produces ethanol [32]. The factor analysis may also support the negative relationship between LAB and mold during the fermentation process. Another negative correlation was detected between NH_3_-N and pH in PC5. In general, therefore, it seems that the pH of silages is closely related to the remaining acid concentration after the neutralization of organic acids by NH_3_-N in the silo. These results are similar to those reported by Bai et al. [44].

As shown in Table 1 and Figure 2, while comparable results were obtained in silages treated with CK and HK for LA content, only comparable results were obtained in silage treated with HK for pH after 45 d of fermentation. Many scholars hold the view that lactic acid-producing cocci, e.g., *Pediococci*, *Streptococci*, *Enterococci*, *Lactococci* and *Leuconostocs*, initiate LA fermentation in the early stages of ensiling and then are replaced by more acid-tolerant *Lactobacilli*, such as *L. plantarum* and *L. brevis* [2,45,46]. From 16S rRNA sequencing, it was indicated that *E. faecium*, *P. pentosaceous* and *L. brevis* were dominant bacterial species among the treated groups at silo opening (Table 3). The most obvious finding to emerge from the 16S rRNA sequence analysis is that synergetic effects of *E. faecium* and *P. pentosaceous* increased LA concentration and decreased pH value during the initial stages of ensiling. A possible explanation for these results may be the enzymatic hydrolysis of lignocellulosic biomass by the cellulolytic potential of *E. faecium* during the ensiling process. A similar LA accumulation and pH decline was also reported by Li et al. [47] for *Pennisetum sinese* (a kind of tropical perennial grass) silage.

In contrast, the monitored aerobic stability of CK with an application rate of 5.7 log cfu g^−1^ by indirect and direct methods was not confirmed with 16S rRNA sequence analysis. A possible explanation of these inconsistent results was mainly related to the growth of undesirable microorganisms, which results in aerobic deterioration. Although the predominant bacterial species were the same in such silage groups, the aerobic stability was different due to different residual WSC, LA, and AA concentrations found in silages.

## 5. Conclusions

This study set out to investigate the impact of alfalfa treated with different kefir sources and their various application doses on fermentation characteristics and aerobic stability. A key finding of the present study was that kefir improved the aerobic stability of silages by inhibiting the proliferation of yeast and mold even with the low WSC content. Factor analysis clearly shows that the antimicrobial activity of kefir has an inhibitory effect on yeast count during aerobic exposure. Considerably more work will need to be done to determine the effects of kefir with an adequate level of carbohydrate sources.

## Figures and Tables

**Table 1:** Some chemical properties of alfalfa silage treated with different kefir sources after 45 d of ensiling.

Item	CON (*n* = 10)	CK (Log cfu g^−1^)			HK (Log cfu g^−1^)			SEM	*p*-Value
		5.0 (*n* = 10)	5.7 (*n* = 10)	6.0 (*n* = 10)	5.0 (*n* = 10)	5.7 (*n* = 10)	6.0 (*n* = 10)		
DM	284.5	305.2	292.3	299.4	272.5	279.5	315.4	9.54	0.151
DM loss	26.2 ^a^	26.2 ^a^	21.0^c^	23.1 ^b^	20.9^c^	25.6 ^a^	26.4 ^a^	0.21	<0.001
pH	5.60 ^ab^	5.70 ^ab^	5.85 ^ab^	5.50 ^ab^	5.90 ^a^	5.45 ^b^	5.55 ^ab^	0.08	0.024
WSC	8.00	6.62	11.68	7.67	8.90	11.30	7.92	1.05	0.607

CON: control, CK: commercial kefir, HK, homemade kefir, DM: dry matter (g kg^−1^), WSC: water-soluble carbohydrate (g kg^−1^ DM), cfu: colony-forming units, SEM: standard error of the mean. The values with different letters (^a, b, c^) in each graph are statistically different (*p* < 0.05).

**Table 2:** Loading vectors of original variables after 45 d of ensiling alfalfa treated with different kefir source ^1^.

Original Variable	PC 1	PC 2	PC 3	PC 4	PC 5
Yeast (after aerobic exposure)	0.780 *	0.514 *	−0.050	0.231	−0.030
Butyric acid	0.780 *	−0.294	0.023	−0.180	0.276
Propionic acid	0.759 *	−0.271	0.120	0.003	0.382
Acetic acid	0.665 *	−0.430	0.429	0.272	0.077
Ammonia nitrogen	0.513 *	0.427	0.446	0.049	−0.547 *
pH	0.345	−0.174	−0.626 *	0.262	−0.538 *
pH (after aerobic exposure)	0.482	0.851 *	−0.016	−0.053	−0.007
Carbon dioxide	0.440	0.846 *	−0.156	−0.091	0.051
Aerobic stability	−0.213	0.750 *	0.340	0.447	−0.061
Water soluble carbohydrate	0.375	0.733 *	−0.288	−0.067	0.240
Mold	0.113	0.416	0.010	−0.630 *	0.366
Mold (after aerobic exposure)	0.223	−0.318	0.751 *	0.410	0.212
Dry matter loss	−0.521 *	0.050	0.693 *	−0.423	−0.145
Lactic acid bacteria	−0.619 *	0.366	−0.088	0.507 *	0.281
Dry matter (after aerobic exposure)	−0.394	0.369	0.228	0.447	0.196
Yeast	0.181	−0.303	−0.826 *	0.335	0.197
Lactic acid/Acetic acid	−0.897 *	0.095	−0.151	−0.218	−0.037
Lactic acid	−0.907 *	0.176	−0.206	−0.068	0.077
Dry matter	−0.815 *	0.018	0.068	0.272	0.124
Eigenvalue	6.428	4.038	2.870	1.885	1.261
Proportion (%)	33.83	21.26	15.11	9.92	6.64
Cumulative (%)	33.83	55.09	70.19	80.11	86.75

^1^ Values estimated by principal factor analysis after Varimax rotation of extracted PCAs * Variables with loading vectors higher than 0.50 were considered to load on specific PCA.

**Table 3:** 16SrRNA sequences isolated from ensiling alfalfa treated with different kefir sources.

Treatments	Silo Opening	After 7 d of Aerobic Exposure
CON	*Lactobacillus brevis*	*Enterococcus gallinarum*, *Enterococcus casseliflavus*, *Weissella paramesenteroides*
CK1	*Pediococcus pentosaceus*, *Enterococcus faecium*	*Lactobacillus plantarum*, *Enterococcus faecalis*
CK2	*Pediococcus pentosaceus*, *Enterococcus faecium*	*Bacillus* sp., *Enterococcus faecalis*
CK3	*Enterococcus faecium*	*Lactobacillus plantarum*, *Lactobacillus brevis*
HK1	*Lactobacillus brevis*, *Enterococcus faecium*	*Lactobacillus plantarum*, *Lactobacillus brevis*
HK2	*Pediococcus pentosaceus*, *Enterococcus faecium*	*Weissella paramesenteroides*, *Bacillus* sp.
HK3	*Lactobacillus brevis*	*Lactobacillus plantarum*, *Lactobacillus brevis*

CON: Control, CK: commercial kefir, HK: homemade kefir, HK1: 5.0 log cfu g^−1^; HK2: 5.7 log cfu g^−1^, HK3: 6.0 log cfu g^−1^; CK1: 5.0 log cfu g^−1^, CK2: 5.7 log cfu g^−1^, CK3: 6.0 log cfu g^−1^.

## Data Availability

Not applicable.
